# Barriers and facilitators to mental health help-seeking for young elite athletes: a qualitative study

**DOI:** 10.1186/1471-244X-12-157

**Published:** 2012-09-26

**Authors:** Amelia Gulliver, Kathleen M Griffiths, Helen Christensen

**Affiliations:** 1 Centre for Mental Health Research, The Australian National University, Canberra, Australia

## Abstract

**Background:**

Adolescents and young adults experience a high level of mental disorders, yet tend not to seek help. Research indicates that there are many barriers and facilitators to help-seeking for young people in the general community. However there are limited data available for young elite athletes. This study aims to determine what young elite athletes perceive as the barriers and facilitators to help-seeking for common mental health problems.

**Methods:**

Fifteen elite athletes aged 16–23 years each participated in one of three focus group discussions. In addition to written data, verbal responses were audio taped, transcribed and thematically analysed.

**Results:**

Participants’ written and verbal data suggested that stigma was the most important perceived barrier to seeking help for young elite athletes. Other notable barriers were a lack of mental health literacy, and negative past experiences of help-seeking. Facilitators to help-seeking were encouragement from others, having an established relationship with a provider, pleasant previous interactions with providers, the positive attitudes of others, especially their coach, and access to the internet.

**Conclusions:**

Intervention strategies for improving help-seeking in young elite athletes should focus on reducing stigma, increasing mental health literacy, and improving relations with potential providers.

## Background

Although the prevalence rates of mental disorders in young people and adolescents (16–34 years, 25-26%) [1] are high, such young people often do not seek professional help [2]. Elite athletes tend to fall within this high risk age group. However, there are very little empirical data available on their experience of mental disorders and subsequent help-seeking [3]. Professionals are an important source of evidence-based treatments for mental disorders [4]. Thus, encouraging appropriate help-seeking by young athletes is an essential preventive and treatment intervention strategy for reducing the prevalence of mental disorders in this group. Such strategies must however, be informed by a clear understanding of the mental health help-seeking issues that are specific to elite athletes.

The limited research which is available suggests that young elite athletes may experience mental disorders at a comparable or potentially lower rate than the general population of young people. A study conducted in the United States found that 21.4% of athletes self-reported clinically significant symptoms of depression [5]. This compares with prevalence rates of 33% in a general sample of college students [6], and 29.2% in community-based young adults aged 18–25 years [7]. The high level of participation in physical activity by young elite athletes may exert a protective effect [8] and thus yield lower prevalence rates of depression in this age group. However, there is currently insufficient evidence to evaluate this possibility. Moreover, a number of factors specific to elite athletes could increase their risk of mental disorders, including sport-related stress [9], living away from home [10], increased levels of risk-taking behaviours associated with drinking alcohol [11], and disordered eating [12], particularly in aesthetic sports such as gymnastics, and in weight-dependent sports such as football and boxing [13,14]. Athletes also suffer from a higher risk of injuries [15-17], especially head injuries, from which depressed mood can result [18-20] and can persist even after retirement from the sport [21].

### Reluctance to seek help

Young people in general often do not seek professional help for mental health problems. A recent Australian study found that only 25% of children aged 4 to 17 years with a diagnosable mental disorder had used any health services in the 6 months prior to the survey [22]. Similarly, a national survey of Australian adults found that only 35% of those with a common mental disorder sought help during the previous year [1]. There are limited studies detailing help-seeking behaviour for mental disorders specifically in elite athletes. Whilst the few available indicate that elite athletes are less likely to seek help than non-athletes, these studies were undertaken 40 years ago and may not reflect the current practices among athletes or non-athletes [23,24]. Although there are no recent studies of help-seeking *behaviour* in athletes, a recent study of elite athletes’ *attitudes* did indicate that athletes may have less positive attitudes towards seeking help than non-athletes [25]. Since attitudes are thought to influence help-seeking in the general population [2,26,27] and this age group is already at risk, young elite athletes may be particularly vulnerable to not seeking help.

### Reasons for not seeking help

#### Young people in general

Many barriers to help-seeking among adolescents and young adults have been reported in the literature (see [28]). These include:

*Poor mental health literacy*[28,29], such as feeling unsure about where to seek help [30], not being able to distinguish between “real distress” and “normal distress” [31], and being uninformed about services available [32].

*Attitudes and personal characteristics* including male gender [2], ethnicity [2], low emotional competence [2,33], negative attitudes to professional help-seeking [2,33], the belief that the problem would go away or could be solved without help [2,30,33], lack of confidence in the professional opinion of the specialist or doctor [34], a culture of self-reliance especially in rural areas [32], not wishing to admit to having a disorder, accessing help making it “real” [31,32], and not selecting GPs as a source of help [34].

*Stigma* including embarrassment [28,30,31,34,35], privacy and confidentiality concerns [34,35] particularly amongst those living in a small town [32], and negative self-perceptions [34].

*Practical barriers* such as lack of transport to access help [36], difficulty obtaining help [30], not enough time [35,37], and financial cost [30,34,38].

Conversely, research has identified a number of possible facilitators of help-seeking, including *emotional competence*[2], *mental health literacy*[28,39], *positive attitudes towards seeking professional help*[28,39], *positive past experiences*[28], *social encouragement*[40], and the *availability of established and trusted relationships with professionals* such as general practitioners [2,40,41].

#### Young elite athletes

It is possible that the barriers to help-seeking by elite athletes are similar to those for young people in general [28,33,42]. However, to date, this issue has not been investigated and there may be particular factors which may influence this process such as the conditions under which athletes live, work, train, and perform that are specifically relevant to this population. The main areas proposed in the literature which may act as barriers among elite athletes are:

##### Attitudes

As noted above, although many young people possess negative help-seeking attitudes, there is evidence that suggests that athletes may have even less positive attitudes towards seeking help from a counsellor than non-athletes [25]. In particular, male and younger athletes have been reported to have less positive attitudes towards seeing a sport psychologist than female and older athletes [43].

##### Stigma

Stigma has been implicated as a barrier to help-seeking in athletes [44,45] and has been the primary focus of recent research with this population. Stigmatisation of athletes who seek psychological services has been documented [44,46], and those who do seek help for mental health problems may be viewed by other athletes and coaches as being weak [25]. For example, a study examining male college athletes’ attitudes demonstrated that they negatively assessed male athletes who consulted a “psychotherapist” but not those consulting a “sport psychologist” [47], the latter being involved more in performance enhancement than mental health issues [48]. By contrast, in a study that investigated these attitudes in female athletes, no negative attitudinal effect was found for female athletes’ attitudes towards other female athletes [48] indicating that males and females may have different perspectives on this issue. Athletes can also be stigmatised by non-athletes. A study of general college students’ attitudes revealed that non-athletes tended to stigmatise male athletes who consulted a sport psychologist for a performance consistency problem, but not those who consulted their coach for the same problem [49]. The stigma associated with help-seeking for mental health problems does not only affect the behaviours of the athletes themselves. It has been reported that stigma often deters professionals working with the athletes from referring an athlete to a mental health professional [50].

One published US study [51] that surveyed college student-athletes found that the top rated reasons for not seeking help from counselling services were ‘no need’, not wanting to experience ‘personal discomfort’, worrying about the ‘perceptions of others’, and a lack of ‘time’. Another US study [52] of the opinions of older (*M* = 53.4 years) retired football players, found that these athletes reported the following as barriers to seeking help: not recognising they had a problem, embarrassment, feeling “weak” if they got help, no insurance, travel and time constraints, and a preference for relying on family and friends, or spiritual means for help. However, to date there has been a paucity of research on young elite athletes’ perceived barriers and facilitators to mental health help-seeking. The current study uses qualitative focus group methodology to explore these issues.

### Aims and scope of this study

This paper reports the results of a qualitative study of the perceived barriers and facilitators to help-seeking for mental health problems in young elite athletes. In this paper ‘adolescents’ refers to those aged between 12 and 17 years and ‘young adults’ to those aged 18 to 25 years [19]. The study focuses on help-seeking for the common mental health problems of depression, anxiety and general emotional distress.

## Methods

Focus group methodology was utilised as it is particularly useful for exploring an issue when there has been little previous research on a topic [53]. Focus groups involve small group discussions on a pre-defined topic. They typically generate large amounts of information that can be subjected to qualitative analysis. The approach capitalises on the interaction between participants to generate richer data than might be obtained from individual interviews [54].

Three focus groups with (*n* = 2, 5, 8) elite athletes were convened and facilitated by the first author (AG), who had adequate knowledge of focus group techniques and no prior relationship with the participants. The focus groups took place between August and November, 2008 at the Australian Institute of Sport (AIS). Ethics approval was sought and granted by both the AIS ethics committee (20080609) and The Australian National University Human Research Ethics Committee (ANU HREC 2008/247). Double cinema passes were distributed to thank participants for their involvement at the conclusion of the focus groups but were not used as an incentive for participation.

### Participants

Participants were 15 elite athletes (nine males, six females) with a mean age of 19.3 years (range 16–23 years) recruited by the distribution of an advertisement flyer to coaches and staff at the AIS. It was necessary to negotiate with AIS coaching staff to find an appropriate time for their athletes to participate. Given the particularly sensitive nature of the issue for these very high profile athletes, participants were not asked if they had previously experienced or sought help for a mental health problem. No participants refused to participate or dropped out of the study after the commencement of the focus groups. Information provided to coaches is provided in Additional file 1: Focus group flyer information.

The AIS is the national sports training institute offering highly competitive scholarships to the most talented young elite athletes from around Australia [55]. Athletes are provided a wide range of facilities and services including meals and accommodation, high performance coaching and training facilities, as well as sports medicine and sports science services, including medical and psychological care. Participants were both Olympic and developmental elite athletes from two different types of sport: one team-based and the other an individual sport. The sport types are not named to protect the identity of the participants.

### Data collection

Each of the three focus groups was of approximately one hour duration and moderated by the primary author (AG) and a research assistant (AP). Only the participants and the two researchers were present during the focus groups. The discussions were structured and based on pre-determined barrier and facilitator topics from the literature (e.g., [28,33]), whilst allowing for other topics to be discussed as they arose.

#### Focus group methods

On arrival, participants read an information sheet and completed a written informed consent form. Participants recorded brief demographic information (age and gender) on a sign-up sheet and wore a name tag to facilitate discussion. Prior to the commencement of the group, participants were instructed by the primary researcher on appropriate focus group behaviour, as well as confidentiality, and the voluntary nature of the discussion. Participants were informed of the facilitators’ research background and professional affiliation, and were advised that the purpose of the group was to engage in a general discussion about help-seeking behaviour for mental health problems in young elite athletes like themselves. To ensure that the participants commenced with an accurate and shared understanding of what was meant by a mental disorder (depression), participants were first presented with a brief vignette, which described a young female athlete “Chloe” who met criteria for both DSM-IV and ICD-10 depression [56]. See Additional file 2: Focus group questions for this vignette as well as the definitions and the full list of focus group questions. Next, in order to gain some preliminary insight uninfluenced by pre-defined topics, participants were asked what they thought were some of the most important mental health issues affecting elite athletes like themselves. Definitions of *seeking help* (“looking for help from a professional source – e.g., a doctor, counsellor, or a psychologist”), *barriers* (“things that can make it harder or stop you from getting help”) and *facilitators* (“things that can make it easier for you to seek help”) were then provided to participants followed by a brief written activity. The first ‘self-initiated’ written activity involved the participants noting down individually on a worksheet provided to them “3 things that might *stop* an athlete from seeking help for a mental health issue”. This activity was an open-ended question designed to generate individual views and thus was conducted prior to the group discussion of barriers. Worksheets for all written activities were collected immediately after completion. Barrier topics derived from the literature on young people and athletes as described above were then presented one by one for discussion by the group. A second brief ‘ranking’ written activity asking individuals to compose a rank order of barriers was then conducted. After enquiring whether there were any additional topics not mentioned an identical process was then repeated for facilitators of help-seeking (“3 things that could make it *easier* to get help for a mental health issue”). Probing questions were utilised to extract further information, and discussions were audiotaped and transcribed verbatim. An additional aim in the present study was to investigate the participants’ views on the appropriateness of a mental health website tailored to athletes. Thus, probing questions in the anonymous help section included the topic of a hypothetical website.

Participants were not provided final transcripts for comment or asked to provide feedback on the findings. One of the researchers (AP) recorded field notes during the focus group. See Additional file 3: Responses to written activities for details of both activities and categorisation of the responses.

### Analysis strategy

Thematic analysis [53] conducted in NVivo 8 was used by the first author (AG) to classify participant statements on reported barriers and facilitators into themes using both a priori and grounded codes [57]. Given that the majority of topics in the focus group discussion were pre-defined, grounded theory [58] techniques for identifying core themes were used only for the self-initiated written activities on barriers and facilitators and the discussion on “mental health issues affecting elite athletes”. However, the themes that emerged from all written activities as well as the volume of discussion created on each topic during the focus groups were both used to determine the major and minor themes for each section of the results. In addition, the self-initiated written activity question responses were categorised into themes and a concept map was created to represent the importance of each theme. The ranked barriers and facilitators in the ranking written activity were reversed scored (where rank 1 = 3 points, 2 = 2, and 3 = 1 point) and tallied so that higher scores indicated higher ranked importance of a barrier or facilitator. Percentages are rounded to the nearest whole or half number. Quotes were selected to best represent the theme discussed. This study adheres to the COREQ checklist for reporting qualitative research [59].

## Results

### Findings

The results of the thematic analysis are presented in three sections below with the first describing the mental health issues facing athletes, the second describing barriers and the third describing facilitators. Within the barriers and facilitators sections, discussion topics are sorted into major themes, and minor themes and then ordered by relative importance as judged by the volume of the discourse created, measured by the number of words and ideas generated for each topic from both the verbal discussion and written activities. Participants are identified by number and gender (i.e., *F1* = female participant number 1).

### Mental health issues affecting athletes

The following is a summary of the themes that were raised during the course of the focus group, either from the initial discussion targeting this topic or during the barriers and facilitators discussion.

#### Major themes

##### Performance

One of the strongest themes to emerge was the issue of performance. The participants felt that feelings of depression and anxiety could result from poor performance – *M1: “I think after, if maybe after a competition you don’t perform very good, you can get depressed and stuff”*.

They indicated that the pressure to perform came from various sources including themselves, their coach, and their families. They also emphasised their high levels of motivation and their perception that they must maintain high standards of behaviour in order to succeed. The athletes indicated that this focus on performance extended to psychologists and they felt that when they were scheduled to see a psychologist they were only able to talk about issues related to sport and performance.

"F1: “When you try to talk about other things, they always relate it back to sport, and they relate it back to goals… even if you want to know about something else.”"

##### Injuries

Another important theme raised by participants was injury and the impact this could have on the individual. Depression, sadness, and anger were all discussed as potential outcomes for both short and long-term injuries. In addition, feeling left out of team activities was raised as a trigger for a change in an athlete’s feelings and behaviour. However, the participants also indicated that motivation to train and to remain a part of their team might assist them in the process of recovering from injury and depression.

##### Athlete appropriate behaviour

The participants felt that an important issue for athletes was the pressure to maintain a high standard of behaviour. They acknowledged that being an elite athlete was a privilege, and that this required them to behave in a certain way – *M2: “You also have the pressures of being an elite sportsperson so you know, you can’t behave like your average person on the street”*. The participants felt strongly that they were very distinct from, and subject to different rules to the general community – *M3: “We are completely different from non-athletes. We have to be more disciplined, and don’t get to release pressure as much”*. Defining themselves as somewhat separate from the community was a theme that recurred throughout the discussion.

##### Weight control

The participants felt that eating and weight control was another important issue for athletes – *F2: “Weight has a big issue in athletes…skinfolds and all that, so yeah I don’t know, some athletes find it hard to deal with that”*. Notably, this was raised primarily by female participants as being an important issue for females in general.

"F3: “I think with girls, it’s like the weight issue. ‘cause it is an issue away from sport and more so when you’re in sport, it’s a bit of a…it’s a high focus point.”"

#### Minor themes

##### Lifestyle issues

Living away from their families was a source of stress, as one participant stated – *F4*: *“When I first came that was the worst thing, like leaving my friends and family behind”.* Balancing their commitments to both their sport and studying were additional sources of stress.

### Barriers

The results for the written activities are presented first, followed by the results for the barrier discussion topics.

**Figure 1:**
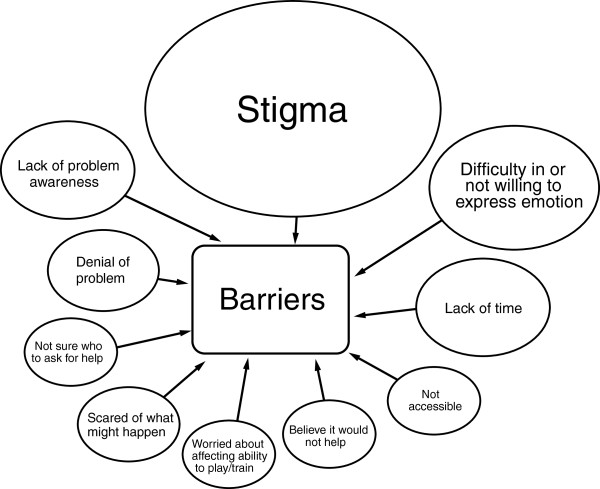
**Barriers to help-seeking for mental health problems reported by elite athletes.** Larger shape indicates a greater number of barriers reported in this topic.

Figure 1 presents a concept map of the barriers to seeking help for a mental health problem for elite athletes reported by the participants in the self-initiated written activity. Overwhelmingly, many responses (18/41, 44%) related to stigma, primarily embarrassment, and the effect of help-seeking on social relationships.

Table 1 presents the top-ranked barriers as reported by the participants in the ranking written activity.

**Table 1:** The top three barriers to help-seeking as ranked by participants in written activity

**Rank**	**Barriers**	**Score**^**a**^
**1**	Not knowing about mental disorders or what the symptoms are	18
**2**	Not knowing when to seek help	18
**3**	Worried about what others will think *[pooled data: coach (7), friends (6), teammates (2), family (1)]*	16

*Note:*^a^ Ranks reverse scored and cumulated across participants. Scores for each topic were calculated by cumulating the reverse-scored ranks (i.e., 1 = 3, 2 = 2, 3 = 1) across participants. Higher scores indicate higher ranked importance.

#### Major themes

##### Public, perceived, personal and self-stigmatising attitudes to help-seeking

The issue of stigma was a primary topic. A common theme across groups was that participants believed that there was a qualitative difference between sport-related issues such as performance anxiety, or goal-setting and other mental health problems such as depression, with the latter being more stigmatised.

"M3: “If it’s performance anxiety and everyone in the team knows their performance has been down then I don’t think it’d be too bad. But if it was like depression or something, then that’d be a bit different”."

The participants were more comfortable seeing psychologists for performance related issues *– F3: “I think if anywhere, it’s more accepted to be able to go and see someon*e ‘*cause it’s something that we have to do”*. However, it was clear that this did not translate into a sense of comfort about seeing psychologists for any other reasons – *F1: “I think most of the time with athletes it’s just like about like goal strategies, and stuff like that, and how to manage nervousness”.*

The participants thought that personality was an important factor in whether the person would feel comfortable seeking help despite stigmatisation of help-seeking – *F1: “Immature people tend to be a bit more like embarrassed to speak about what they’re feeling”* and *M3: “I’d say personality again…’cause I don’t care, but I know that other people that are seeking help would care”.* The participants agreed that athletes would be worried about others finding out if they were seeking help for a mental health problem. Many of the comments related to the demands of being an elite athlete and being concerned that others would think they were not coping effectively or were *“*weak*”*. One of the participants remarked *–*

"F4: “You don’t want them to think that you’re not handling the pressure…That’s the thing with athletes, like you’re not really supposed to show your weaknesses kind of thing, ‘cause that like lets your competitors know, so that’s why a lot of the time you wouldn’t go see the psychologist or whatever, just ‘cause that becomes your weakness.”"

The athletes had differing views about who they would most be concerned about finding out that they were seeking help. Their coach was a much greater concern for older athletes than for younger participants. Teammates (and friends who were teammates) were also high on the list of those whom the participants would worry about – *F2: “My coach and probably team mates,* ‘*cause we’re surrounded by that”*. Participants also thought it could be worrying for athletes if their parents and family found out. Additionally, they were concerned about negative consequences of the social aspect of their friends finding out if they were to seek help. However, some of the participants thought this could be viewed as being a positive thing if it were close friends – *F2: “Some people wouldn’t probably mind their close friends knowing* ‘*cause then the support network they could build to help them get through it would be good”*. When asked what athletes thought of other athletes seeking help, the responses were supportive and non-judgmental – *F2: “I think it’s good if they’re getting help and they’re dealing with what the problem is, then it’s good for them”.* However, when prompted, many of the participants thought that confidentiality with help-seeking was very important and they rarely, if at all heard about other athletes seeking help. Despite the apparently accepting attitude of the majority of their peers, many participants were very reluctant for anyone to know if they themselves were to seek help.

"F2: “It just makes it worse for the athlete that needs the help, and maybe it would just make them shy away from, them thinking they have a problem, like pretend that there’s nothing wrong ‘cause they don’t want people looking at them differently.”"

Participants agreed that the media had a large role in determining what the public would think of an athlete seeking help for a mental health problem. Overwhelmingly, the media was thought to exaggerate and exacerbate the issue for them, and make the individual *“feel worse about their problem…Instead of focusing on the positives of them trying to get help, it’s just them putting them down all the time” (M4)*. Others felt that when well-respected athletes actively chose to publicise their help-seeking for mental health problems, that this could be received in a positive way, and could also be useful for athletes.

"F2: “I think it makes…other people in the world that aren’t athletes realise that there’s nothing that’s different between us superstar athletes, as they might put our names to - you know, we’re all the same.”"

##### Lack of knowledge about mental health services

The participants considered that lack of knowledge about the services available and how to access them could act as a barrier to seeking help. However, they thought that this would primarily affect athletes living away from a centre like the AIS, who may not have direct access to services – *M5: “If you were living at home you wouldn’t really know where to go”*. Participants agreed that a lack of knowledge about what might happen in a consultation could act as a barrier for an athlete seeking help. Most thought that feeling worried about being uncomfortable approaching someone for help, or being frightened about not knowing what to expect could act as a barrier. The participants also thought that some athletes might feel anxious about what they expected would happen during a session with a counsellor or psychologist – *F2: “Some people may find it hard to sit just one on one with someone, ‘cause it’s awkward or uncomfortable for them…it’s sometimes hard to talk about your own weaknesses”.* They felt that some athletes may be concerned that a health provider would not understand their problem – *M3: “I think sometimes you don’t know whether the other person is going to understand or not”,* particularly if it was the athlete’s first visit. The athletes also indicated that they were uncertain about when it might be appropriate to see a professional *– F4: “Well sometimes you don’t know what to say, like you can’t just go up to a counsellor and say, well I’m a bit sad”.* The participants were not sure which professionals were appropriate for mental health problems.

"F4: “The psychologists here are sport psychologists, sometimes things that happen away from sport you’re not sure whether you can go and see them about that, or is that an issue for a counsellor.”"

However, they were unanimously adamant that general practitioners were not an appropriate first source of help for mental health problems.

"M6: “I always thought of doctors as like second referral, like you go to a counsellor or a psychologist and then you go to a doctor after that, if they think you should, or if it’s not working.”"

##### Lack of knowledge about the symptoms of mental disorders

The participants considered that lack of knowledge about the symptoms of mental disorders was an important barrier to seeking help for athletes. They raised the point that depressed mood and *“up and down emotion” (F1)* was *“an everyday occurrence” (M3)* for elite athletes in their position. They explained that they experienced regular physical strain and found it difficult to distinguish between the fatigue caused by this physical exhaustion and depression or anxiety.

"F2: “Being an elite athlete isn’t easy…you’re pushing your body to extremes almost every day and sometimes you do get depressed and, or upset or down because you’re always almost totally fatigued every day. So sometimes, I guess…you can blame your depression and anxiety and that on fatigue, when it might not be the fatigue that’s creating the depression or the anxiety. But you could put a cover on it like that, saying it’s from training.”"

The participants acknowledged that athletes might find it difficult to apply their knowledge of the symptoms a depressive or anxiety disorder to themselves. They might be knowledgeable about the symptoms of these mental disorders, but not know if what they were experiencing was *“just a feeling” (F1)* or indicative of a mental disorder – *F4: “Maybe it’s not so much like, you don’t know about mental disorders but you don’t realise that you might have it”.* This also applied to eating disorders. However, according to the athletes, eating disorder differed from anxiety and depression in that that the athlete experiencing the symptoms might not be aware that they had any sort of problem at all, even when others around them clearly perceived the problem.

"F2: “They’ll just keep going on with what they’re doing thinking that it’s right, but the people around them can see that it’s not, so they might not even know that they’ve actually got a problem.”"

This theme of having someone else recognise the problem before the individual was common. Coaches and other people close to the athlete were viewed as being in a position to see the need for help in some cases – *M3: “I went to get help after the coaches told me”.*

##### Negative past experiences

The participants felt that past experiences could act as a barrier to future help-seeking. They had access to free counselling with certain providers. However, a problem in relating to this provider in the initial consultation could act as a barrier to them returning to this person for help.

"F1: “My coach always used to make me try and go see [a certain psychologist], and I didn’t like it, and so I’d sit in there for the whole session and I wouldn’t want to say anything, and just ‘cause she was really like, not the type of person that I wanted to talk to.”"

The participants thought that this could even hinder them from seeing a different person for help *– F2: “Yeah, definitely if you had a bad experience you wouldn’t want to try it again with someone else”.* It was thought that a close relationship with the provider would act as less of a barrier to future help-seeking. The greatest barrier to future help-seeking was a breach in confidentiality *– F2: “If you lost your trust in someone, and they said something to someone else”.*

#### Minor themes

##### Lifestyle factors

There was some disagreement about whether lack of time was a barrier to seeking help. Notably, the younger participants indicated that if you really needed help, you could *“always find time” (M3)*. However, the older participants considered that time constraints were a significant problem – *F2: “If you do have the time you just want to be resting. ‘Cause you sometimes think that the fatigue is creating what - the depression or whatever”.*

As part of their scholarship, AIS athletes are provided with access to a range of services including psychologists on location at no personal cost. Considering these arrangements, the participants felt that not having enough money or transport to seek help was not a major issue for elite athletes in their position. They did acknowledge however, that it would be more difficult for lower level or more isolated athletes who did not have easy access to cost-free services – *M6: “If it wasn’t free, I wouldn’t go”*.

##### Personal characteristics

The participants agreed that gender would definitely be a barrier for athletes, in that males would find it harder to seek help than females. They thought this could be because males perceive seeking help as a *“sign of weakness” (M4)* or as an act which lowers their *“social status” (M4)*, and they are less able than females to articulate their feelings. Age was also thought to be an important barrier, although some participants believed that younger athletes would be less likely to seek help, and others indicated that older athletes might be more confident and perceive themselves as better able to manage their own problems.

#### Facilitators

The results for the written activities are presented first, followed by discussion topics. Facilitators were generally discussed in much less detail than barriers, and the participants generated more diverse responses.

**Figure 2:**
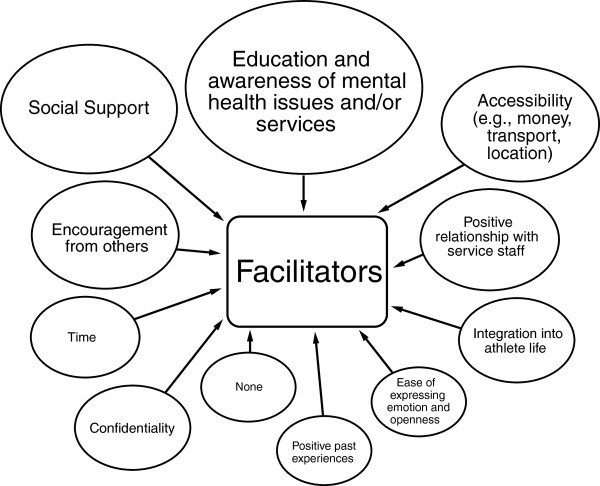
**Facilitators of help-seeking for mental health problems reported by elite athletes.** Larger shape indicates a greater number of barriers reported in this topic.

Figure 2 presents a concept map of the facilitators for elite athletes in seeking help for a mental health problem as reported by participants in the self-initiated written activity. The largest number of facilitators were reported in the topic of education and awareness of mental health issues and services (10/45, 22%).

Table 2 presents the top-ranked facilitators as reported by the participants in the ranking written activity.

**Table 2:** The top three facilitators as ranked by participants in written activity

**Rank**	**Facilitators**	**Score**^**a**^
**1**	Already knowing a health professional quite well (e.g., counsellor, doctor)	24
**2**	Being aware of your feelings and finding it easy to express them	19
**3**	Others have a positive attitude towards seeking help *[pooled data: coach (9), friends (4), teammates (3), family (1)]*	17

*Note:*^a^ Ranks reverse scored and cumulated across participants. Scores for each topic were calculated by cumulating the reverse-scored ranks (i.e., 1 = 3, 2 = 2, 3 = 1) across participants. Higher scores indicate higher ranked importance.

#### Major themes

##### Encouragement and the positive attitudes of others

Participants differed in their views about whether it would be useful for others to encourage an athlete to seek help. Some viewed it positively, particularly if it was the coach offering advice. Notably, participants thought that their coach could act as a gatekeeper to services – *F1: “Sometimes if there’s a problem you sort of talk to your coach, and maybe then they’ll tell you if you need some more help or something”.* Additionally, they thought that it needed to be dealt with very sensitively and only by someone close to the athlete. They felt it would be acceptable if the encouragement organically arose from a discussion with a person close to the individual who was respected and trustworthy.

"F1: “If you’d like been talking about issues, but you hadn’t really discussed about getting help, and then someone would say to you, like do you think you should get help then that would be ok, I think, but not for someone to say to you – ‘you need help’”."

Despite this, the participants felt that the decision to seek help was ultimately up to the individual and that encouragement would not necessarily be useful unless the person wanted to seek help.

The participants generally agreed that it was important that others around the athlete including their family, coach and friends had positive attitudes towards seeking help, and that this was an important facilitator – *M7: “probably the biggest one I think”*. Some thought it would not be helpful if their coach knew – *F4: “I don’t think it would help, like you still feel a bit uncomfortable if they know”*. Others believed it could be very helpful if their coach knew and had a positive attitude towards seeking help – *M1: “Coach is probably the one that’s going to have the most influence over you”*.

##### Established relationship with provider

The majority of participants felt that having an established relationship with a health professional would act as a facilitator to them seeking help. Overwhelmingly, they thought that knowing the psychologists they would be accessing made it *“easier if you need help” (M4)*. However, other athletes acknowledged that knowing the counsellor might only be important for some athletes, in that some people would feel more comfortable with professionals regardless of any previous relationships.

#### Minor themes

##### Access to the internet and online mental health services

Generally, the participants felt that anonymous access to the internet may act as a facilitator for a small minority of athletes who may not feel comfortable approaching a health provider in person – *F2: “I think that’s good for people that find it hard to I guess talk one on one”.* The participants felt that the internet facilitated access to information and services, but had concerns about their quality. They felt that a small minority of athletes may want to use online mental health services on the internet, yet that most athletes would have enough confidence to talk to someone face to face – *M4: “Depends on the person, ‘cause some people would want to speak face to face, while others find that way easier”*.

##### Emotional competence

Participants had differing views about whether awareness of feelings played a significant role in facilitating an athlete to seek help. Some thought that it was useful in seeking help – *F1: “Well you have to know what’s wrong with you to talk about it, you need to be open to talk about it as well”.* However, others believed that personality and openness of feelings would be more useful in assisting them during a psychologist or counselling session, rather than in the act of seeking help.

##### Characteristics of provider and support staff

Most participants thought it was of little importance for the support staff such as receptionists to be friendly. Yet it was considered important for the provider themselves to be friendly – *M3: “for the counsellor it’s very important…you wouldn’t want to deal with them if they’re not”.*

## Discussion

### Mental health issues affecting athletes

The participants raised several themes which have previously been discussed in the literature on elite athletes, including performance and sport related stress [9], injuries [15-17], weight control and disordered eating patterns [12-14], and living away from home [10]. An important theme underlying all of the focus group discussions was the participants’ perception that athletes were very different to general members of society. This was highlighted by the view that participants were expected to behave differently from and to be more disciplined than members of the general public (they cannot behave like ‘the average person on the street’). This perception that they are subject to different rules may have implications for all their behaviours including help-seeking.

### Barriers

The most striking finding was the dominance of stigma as a barrier to athletes seeking help for mental health problems. Over 40% of the barriers listed by participants related to stigma and the embarrassment an athlete would feel in seeking help. Additionally, it was noted during the groups that the discussion on stigma was clearly an important topic for the athletes, and was substantial in length. Although this was partly attributable to the larger number of questions on this topic due to the focus on stigma in the literature, it was also a consequence of the lengthy and animated nature of the participants’ discussion of the issue.

The focus group discussion suggested that the athletes may have high levels of self-stigmatising attitudes [60]. As is consistent with previous research on the stigmatisation of help-seeking in athletes [47], the participants considered it more acceptable for an athlete to see a psychologist for performance or goal-setting reasons. They considered it would be much more embarrassing for the athlete if they were to seek help for depression, or any other *“major” (F1)* concern not related to sport.

The athletes also demonstrated high levels of perceived stigma (a perception that others hold stigmatising views) [60]. They were most concerned about people connected with their sport finding out if they were to seek help as it may be perceived as a sign of their *“*weaknesses*”* or inability to cope, and expose them to the negative views of others. Their coach, team mates, and their competitors were all cited as people they would not want to find out if they were to seek help for a mental health problem. However, some participants thought that disclosure to friends could be useful as the support network of their close friends could help them through their problem. Friendship networks often replace the support network of the family when the athlete is required to move away from home [10]. However, when explicitly questioned about what they thought of other athletes seeking help, they appeared very accommodating of this behaviour in others. This may reflect social desirability [61] or a genuinely lower level of stigmatising attitudes to others, whilst retaining a strong sense of self (or internalised) stigma [62].

It was evident that the participants strongly disapproved of the media’s portrayal of athletes with mental health problems. In general they felt that the media determined what the public thought of athletes, and tended to exaggerate mental health problems experienced. Additionally, media articles often claim that the public see athletes as immune to the challenges facing members of the general community [63,64], which may place additional pressure on the athletes to avoid identifying potential mental health problems. Despite this, the participants saw the value of an athlete telling their story, not only for other athletes but also to help the public understand that they, like anyone can develop mental health problems.

The prominence of stigma as a barrier to help-seeking in athletes is consistent with previous reviews on young people in general [28,33]. It is clear that stigma is an important barrier to seeking help in athletes and may be even more influential in this group than in the general community due to the athletes’ perceived attitudes of the media and the general public. It may be helpful to implement programs for young elite athletes that can reduce the level of stigma surrounding common mental disorders [65], which might in turn lead to increased help-seeking in this group. It might be equally or more important to promote help-seeking among athletes by providing evidence-based online programs that can be accessed anonymously.

A lack of knowledge about symptoms of mental disorders was considered a major barrier to seeking help in the present study. The participants considered it particularly problematic to apply their knowledge of the symptoms of mental disorders to themselves, and to determine whether the symptoms they experienced were severe enough to require professional attention. This issue has also been reported in research involving young adults in the community [31]. A key finding was that the athletes found it very difficult to determine the difference between normal feelings of tiredness and sadness associated with their sport, and symptoms of a possible mental disorder. This supports Schwenk’s [66] assertion that athletes may be more susceptible to misdiagnosis, especially for problems related to training and performance as their symptoms may be viewed from a physiological perspective. In this case it is the athlete themselves who may misdiagnose their problem. Schwenk [66] further argues that there are strong similarities between overtraining or “a negative response to training stress” [44], and depression, which could also result in under-diagnosis by professionals of depressive disorders. The participants in the present study reported that eating disorders may remain undetected by the athletes. Low body fat levels are often required for participation at an elite level and this may contribute to an athlete’s perception that their eating disorder is acceptable or even ‘normal’. As such, the participants proposed that the athlete’s support network was an important means of providing perspective on this issue and helping the athlete see that they may have a problem. This view is consistent with the conclusions of a review of the research by Rickwood et al. [33], which highlighted the importance of social influences on help-seeking.

Whilst they acknowledged that some athletes similar to their peers in the community [30,32], may not know about mental health services, this particular sample of athletes felt that they knew about accessing available services. However, they did believe that not knowing what to expect during a visit could be a significant barrier for athletes. This fear of the consultation itself has also been found in previous research on young people [33,35,67]. In particular, a lack of knowledge about when it might be necessary to access these services was highlighted by the comment expressing that it may not be appropriate to seek help from a counsellor for something like feeling only *“a bit sad”.* The athletes also did not believe that a general practitioner was an appropriate place for seeking help for mental health problems, a finding that is consistent with prior studies involving young people [32,68]. Finally, in accordance with the literature on community-dwelling youths [34] the participants believed that athletes might be concerned that a health provider would not understand or might think negatively of them. This lack of knowledge about mental disorders and their treatment has important implications for the continued refinement and delivery of programs that improve mental health literacy both for young people in the general community [69] and among young elite athletes [65].

The athletes in the present study considered that males would have more difficulty seeking help than females, a finding in agreement with previous research involving young people in the community [2,40]. However, their opinions for age were less clear. The participants had differing views on whether older or younger age could be a barrier. In the literature, younger aged athletes have been found to possess more negative attitudes to seeking help [43]; thus, attitudes could act as a barrier for younger age groups. Nevertheless, the participants considered immaturity irrespective of age to be important. Previous literature has demonstrated that more mature young people, especially females are likely to have higher levels of emotional competence, which is associated with seeking help [30,40].

As described in reviews of help-seeking among young people in general [43], the athletes considered that negative past experiences could act as a barrier to future help-seeking. This opinion was informed by their personal experiences involving unsatisfactory contacts with potential sources of help. They particularly thought it important that the provider adhered to confidentiality principles, and observed that failure to do so would serve as a serious deterrent to help-seeking, which is consistent with previous research using a community sample of young people [32,34,43].

The athletes were divided as to whether time constraints were a significant barrier to seeking help. Generally, the younger participants (16—17 years of age) were less likely to consider lack of time a problem. Given, the likely workload of older young athletes (e.g., schoolwork, university and other commitments), this is an expected result, particularly as time factors are generally endorsed as a barrier to help-seeking in studies with adults [70,71] and older (*M* = 19.1 years) college student-athletes [51]. The participants felt that money and transport were not relevant to them, though they acknowledged this may be a problem for more isolated athletes who do not have access to facilities. This contrasts with the findings from prior research on young people for cost [30,34] and transport [36]. However, this is unsurprising given the cost-free facilities available to the participants in the present study.

### Facilitators

Generally, the athletes believed that encouragement sourced from trusted close relationships, the positive attitudes of others, and a good relationship with a mental health provider would facilitate help-seeking.

The athletes thought it was very important to help-seeking to have an established relationship with an appropriate provider, in their case a psychologist. This is consistent with reviews of help-seeking in young people [33]. Considering that *positive relationship with providers*, only represented 9% of the facilitators reported prior to the focus group discussion, and *already knowing a health professional quite well* was ranked first after the discussion, it appears that the participants found this relationship more important after discussing it with their peers. Considering they may potentially need to see a psychologist regularly particularly for performance issues, this may to be an especially important facilitator for athletes. Given the importance placed by the athletes on the establishment of a relationship with a provider, it seems important that providers of mental health care develop a rapport with the athletes, even if this is outside professional consultations. The athletes believed the characteristics of the actual provider themselves was vital, a view which is consistent with previous qualitative research investigating adolescents’ views on provider characteristics [41,72]. This also relates to the impact of negative past experiences [39,40], in that the positive nature of previous encounters with the provider is important.

Consistent with previous research involving non-athletes [40], the participants believed that athletes would consider it very important for those around them to have a positive attitude towards seeking help. In particular, they thought it was vital that their coach had a positive attitude, and this was also reflected in the ranking of facilitators, where coach clearly outranked all other sources including friends, family and team mates. A previous Australian study implemented an educational program for football coaches in a rural community setting [73]. Data from the current study suggest that such programs should be made available to elite sport coaches to enhance their understanding of mental health problems and needs and to assist them to facilitate help-seeking among elite athletes. The athletes in the present study also considered it important to normalise mental health problems as well as seeking help, a suggestion that is consistent with the findings of a previous study of young people [39]. The athletes felt that encouragement to seek help by others could only be effective if it came from a highly trusted source, such as the coach or a close friend or family member. Encouragement to seek help from a person close to the individual with mental health problems has been reported in past reviews and research as a facilitator of help-seeking among young people [33,40]. However, the athletes emphasised that the source must be a trusted individual, and even then, the issue must be dealt with sensitively.

Participants in the present study believed that online resources might be useful for those athletes not comfortable with face-to-face contact. This is consistent with prior research indicating certain groups may be more comfortable with internet help-seeking [74]. They also considered that an online resource from a trusted source, which primarily assisted them to determine whether they should seek help, could be useful for all athletes.

The athletes had differing views about whether emotional competence was a significant facilitator of seeking help. Some felt that it was important, whereas others thought it may only facilitate the consultation itself, rather than the initiation of help-seeking. The latter view contrasts with the findings from previous reviews [33] indicating that emotional competence is an important factor in help-seeking.

### Limitations

A primary limitation of this study is the modest sample size. Given the schedules of these extremely busy athletes, recruitment was difficult. However, the key themes described emerged in each successive group. Participants were largely self-selected or invited by their coaches to participate and few sports were represented in the focus groups. Thus it is possible that important barriers and facilitators were not identified in this study.

Another potential limitation is that the structured nature of the focus groups might have influenced the amount of discussion produced on each topic. We attempted to address this in the study design by the inclusion of the self-initiated written activity. The provision of a depression vignette was intended to inform the discussion of the focus groups. However, we acknowledge that the vignette may have influenced the participants’ responses to some extent.

A final limitation is that the thematic analysis was conducted by one researcher. However, it has been suggested that a single coder can generate valid interpretations of the data provided that the process followed is methodologically rigorous [53].

## Conclusions

To the authors’ knowledge, this is the first qualitative study of both the barriers to and facilitators of help-seeking in young elite athletes. Despite its limitations, the study provides valuable information about mental health issues specific to young elite athletes, as well as a preliminary insight into the barriers and facilitators to help-seeking in this group. Its results indicate that whilst young elite athletes feel ‘different’ to their peers in the community, they reported similar barriers and facilitators to help-seeking to those reported in the literature for young people in general. Barriers included stigma, a lack of mental health literacy, and negative past experiences, and facilitators included positive attitudes from peers, and positive relationships with providers. These findings suggest several approaches for increasing help-seeking amongst young elite athletes. An initial strategy would involve providing programs to young elite athletes that are specifically designed to reduce the stigma associated with mental illness and mental health help-seeking in athletes. A second approach would be the provision of tailored materials that will increase the young athlete’s mental health literacy, and in particular to increase their knowledge of their own symptoms and their awareness of where they can access anonymous, evidence-based online mental health programs. There is also a need to develop techniques for engaging and training coaches to facilitate appropriate help-seeking among their athletes. Finally, given that many elite athletes have access to an on-staff mental health provider such as a psychologist, it is important for sport and athletic organisations to encourage and actively facilitate a positive relationship between the athletes and their mental health staff.

## Competing interests

The authors declare that they have no competing interests.

## Authors’ contributions

AG conducted the study, carried out the data analysis and wrote a draft of the manuscript. KG and HC supervised all stages of the research, and contributed to the design of the study, the analysis and editing, and commented on the paper. All authors read and approved the final manuscript.

## Pre-publication history

The pre-publication history for this paper can be accessed here:

http://www.biomedcentral.com/1471-244X/12/157/prepub

## Supplementary Material

Additional file 1 Focus group flyer information. Click here for file

Additional file 2 Focus group questions. Click here for file

Additional file 3 Responses to written activities. Click here for file
