# Supplementary material for: Barriers and facilitators to mental health help-seeking for young elite athletes: a qualitative study
Source: BMC Psychiatry. 2012 Sep 26;12:157. doi: 10.1186/1471-244X-12-157 (PMC3514142; doi:10.1186/1471-244X-12-157)
Supplement: Additional file 1 — Focus group flyer information. [file 1471-244X-12-157-S1.doc]

**Focus group flyer information**

**Focus Group Study**

- The Australian National University (ANU) and the Performance Psychology Department are conducting some short focus groups with athletes at the AIS.
- We would like to find out their opinion about why athletes may or may not seek help for mental health problems such as depression or anxiety, as well as the types of services they prefer.
- No mental health screening will take place.
- This research will be used to create an intervention targeted at increasing rates of help-seeking in athletes, which could lead to optimum mental health and enhanced performance for more athletes.
- Focus groups involve an informal discussion amongst 6 -10 athletes lasting about 1.5 hours. Their participation in this project would be voluntary, and they can withdraw from the study at any time.

*Note: Contact details for the AIS researchers were also provided.*
