# Supplementary material for: Barriers and facilitators to mental health help-seeking for young elite athletes: a qualitative study
Source: BMC Psychiatry. 2012 Sep 26;12:157. doi: 10.1186/1471-244X-12-157 (PMC3514142; doi:10.1186/1471-244X-12-157)
Supplement: Additional file 2 — Focus group questions. [file 1471-244X-12-157-S2.doc]

**Focus group questions**

**Depression vignette:**

“Chloe is an 18 year old girl living in the AIS residences. For the last four weeks, she has been feeling unusually sad. She has always loved playing netball, but doesn’t seem to care about it anymore and lately has been trying to avoid training sessions. She has lost quite a bit of weight because she is not eating properly. She seems to be finding it difficult to concentrate and make normal day to day decisions. This is having a negative effect on her training and school work. She also seems to be very tired and run down. Friends, teachers and her coach are all concerned about her” (adapted from Kelly, 2006, [56]).

**BARRIERS**

**Definitions:**

*Seeking help*: Looking for help from a professional source – e.g., a doctor, counsellor, or a psychologist.

*Barriers*: Things that can make it harder or stop you from getting help

**Questions:**

As an elite athlete, do you think it would stop you from seeking help if…

- You don’t know much about mental disorders or what the symptoms are?
- You don’t know when to seek help?
- You don’t know how or where to seek help?
- You don’t know what to expect in a doctor’s appointment or counselling session?
- You are of a certain gender, culture, religion or age group?
- You feel like you don’t have enough time?
- You haven’t got money, or transport?

*In addition, perceptions were sought on the following topics related to stigma:*

- Would it be embarrassing for an athlete to be seen asking for help?
- Do you think it would be worrying for an athlete if their coach/teammates/friends/ family heard that they were seeking help?
- What do athletes like yourselves think of other athletes who get help for mental health problems?
- What do you think the public think of an athlete who gets help for mental health problems?

**FACILITATORS**

**Definitions:**

*Facilitators*: Things that can make it easier for you to seek help

After this, a third written activity asked participants to list three “things that could make it easier” for an athlete to seek help for a mental health issue. Again, this activity was conducted before the group discussion of facilitators to obtain the athletes’ responses independently of the group responses. A list of facilitators was then presented one by one for group discussion:

As an elite athlete, do you think it would it make it easier to seek help if:

- You are more aware of your feelings and find it easy to express them?
- You already know a health professional quite well (e.g., counsellor, doctor)?
- Your friends, family and coach all have a positive attitude to seeking help?
- Other people encouraged you to seek help?
- The staff (e.g., receptionist, doctor, counsellor) are friendly and approachable?
- All athletes were required to see a counsellor or psychologist as part of their preparation and training?
- Your access to information and help is anonymous (e.g., the internet) ? –

*Important prompts in this topic were*

- Is the Internet somewhere you would want to go for help for a mental health issue?
- Would an anonymous website designed specifically for athletes be useful?
- What sort of information on this website do you think would be most useful?
- How do you think we could get young athletes like yourselves interested in using a website like that?
- If there was an online treatment program available that you could do yourself, do you think athletes might be interested in using it?
