# Supplementary material for: Barriers and facilitators to mental health help-seeking for young elite athletes: a qualitative study
Source: BMC Psychiatry. 2012 Sep 26;12:157. doi: 10.1186/1471-244X-12-157 (PMC3514142; doi:10.1186/1471-244X-12-157)
Supplement: Additional file 3 — Responses to written activities. [file 1471-244X-12-157-S3.doc]

**Responses to written activities**

### Barriers

Participants’ original spelling has been preserved. One participant chose to verbalise their comments and this is marked (verbal).

**ACTIVITY 1**. Before discussion on barriers.

Instructions for participants were: Please write down 3 things that might **stop** an athlete from seeking help for a mental health issue.

**A: Stigma**

*Embarrassment*

1. Embarrasement [sic]
2. Embarrassment
3. Embarresed [sic]
4. Feeling embarrassed about accessing services
5. Embarrassment – don’t want anyone to know about it
6. Embarrassment – feeling that it may be made out a bigger issue than what it really is
7. Being embarrassed about thinking they have a problem
8. Embarrassment – you don’t want people to think you can’t handle the pressure etc.
9. If they are worried about being judged or people finding out

*Effect on social relationships*

1. Social status
2. Social view
3. Social status
4. What other people think
5. Pride
6. Macho-ism
7. That if you seek help you are different from others
8. Pressure from others around you

**B: Difficulty in or not willing to express emotion**

1. Find it hard to talk about, not being confident in what people might think
2. Find it hard to talk about
3. Want to keep issues to yourself
4. Not like to talk about issues so they may keep to themselves
5. They don’t feel comfortable to confront someone about it
6. Feel like they don’t have anyone to talk to about it

**C: Lack of problem awareness**

1. Athlete being aware that they may need help
2. Lack of awareness that there is a problem
3. Not know if you have a problem or you are being a drama queen
4. You may not realise you need it e.g., your [sic] so focussed on a goal you don’t realise you need help in other areas

**D: Time**

1. Avilibility [sic] of doctors & your time
2. Availability – Can’t fit it into their schedule
3. Lack of time
4. Time due to busy schedule

**E: Denial of problem**

1. They don’t actually think anything is wrong
2. Denial of not being a sufferer
3. The thought that you don’t need help

**F: Scared of what might happen**

1. Scared
2. Scared they actually might have something wrong

**G: Accessibility**

1. Not accessible

**H: Worried about affecting ability to play/train**

1. Ability not to train

**I: Think it would not help**

1. Issue of not thinking a counsellor could help

**J: Confidentiality**

1. Feeling that the information given may get back to someone i.e., confidentiality

**K: Not sure who to ask for help**

1. Not aware of who you should speak to

**ACTIVITY 2**. Ranking activity after discussion on barriers.

Instructions for participants were: Please rank the top 3 barriers below by numbering them (1, 2, 3) in order of those you see as the most important in stopping an athlete from seeking help for mental health issues.

| Barrier | Rankings | TOTAL |
| --- | --- | --- |
| **Not knowing about mental disorders or what the symptoms are** | 1 1 1 3 2 1 1 | **18** |
| **Not knowing when to seek help** | 2 2 2 3 2 1 1 1 | **18** |
| Not knowing how or where to seek help |  |  |
| Not knowing what to expect in a doctor’s appointment or counselling session | 3 3 | 2 |
| Gender | 3 | 1 |
| Culture |  |  |
| Religion |  |  |
| Age group | 3 2 | 3 |
| Not having enough time | 1 3 2 2 3 | 9 |
| Location of Services | 1 |  |
| Transportation | 3 | 1 |
| How you feel about yourself seeking help | 1 2 3 | 6 |
| Worried about what your coach will think | 2 1 2 | 7 |
| Worried about what your teammates will think | 2 | 2 |
| Worried about what your friends will think | 3 1 2 | 6 |
| Worried about what your family will think | 3 | 1 |
| ***Worried about what others will think (pooled data: coach, teammates, friends, family)*** |  | **16** |
| Other (please explain)  *Trusting your psychologist* | 3 | 1 |

***Scoring:*** 1=3, 2=2, 3=1, numbers added for total score; Higher scores = more higher ranked votes. Note: Sheets filled out incorrectly, and any ranks over 3, were omitted. On 2 sheets, ranks were included for all facilitators but not in order, e.g., five were given a 1, four were given a 2, and 1 was given a 3.

### Facilitators

Participants’ original spelling has been preserved. One participant chose to verbalise their comments and this is marked (verbal).

**ACTIVITY 3**. Before discussion on facilitators.

Instructions for participants were: Please write down 3 things that could make it **easier** to get help for a mental health issue.

**A: Education and awareness of mental health issues and/or services**

1. Discussion groups, making athlete’s more aware of any potential mental health issues they may have
2. Education
3. Education
4. Education
5. Knowing that health services are available
6. Knowing where to find the right help
7. More knowledge on mental health issues
8. Knowledge
9. Have people come in and do talks about how they went through it themselves
10. And knowing that you have a problem

**B: Social Support**

1. Support from family and friends
2. Support
3. Support
4. Support
5. Support from coaches & other support staff
6. More openness about issues within teammates

**C: Accessibility (e.g., money, transport, location)**

1. More readily available eg. location + price
2. It’s free – got nothing to lose by going – Except 30 min!
3. Close. Easy to get to
4. Psychs coming to you & follow-ups on previous visits
5. Having a psychologist readily available
6. No cost

**D: Encouragement from others**

1. Just being told that you need the help, should be enough
2. Having a coach recommend/push you to go see one
3. Being encouraged by family, friends, teammates etc. to get help
4. Coaches/friends/family giving you advice for you to go see someone
5. Someone telling you how much it helped them
6. Having talks set up for…people that choose to go to them of people that have gone through stuff like what they might be going through and how they got help and how they dealt with it (verbal)

**E: Normalising seeking help**

1. If a lot of people/it was common to go
2. If you went regularly – not just for issues
3. Make it a comfortable
4. Making it a comfortable situation for the people to think it’s ok to go and see say a doctor for that or, or the psychologist not just because it relates to your sport and your racing but you’re dealing with something that helps (verbal)
5. Making it...what they’re going through isn’t…weird or…unusual. Like so they haven’t got a disease or anything…try and making it a comfortable situation for them to deal with, so… if they wanted to go and see someone about it, that it’s kind of openly out there, that it’s ok for them to see these people…Dumbing it down for the athlete so they don’t feel like they’re singled out (verbal)

**F: Positive relationships with providers**

1. Know someone that your happy to speak to
2. More variety of psychologists
3. If you had a good relationship with your counsellor/psychologist
4. Having trust and comfort within your psych

**G: Confidentiality**

1. Knowing that it is all confidential
2. Being confidential

**H: Ease of expressing emotion and openness**

1. Being an open person & willing to talk.
2. Your happy for people to know your talking about stuff

**I: Time**

1. More time to see one
2. Allowance for time to go see someone

**J: Good past experiences**

1. Good past experiences

**K: None**

1. I don’t think there is anything that would make it easier to get help

**ACTIVITY 4.** Ranking activity after discussion on facilitators.

Instructions for participants were: Please rank the top 3 facilitators below by numbering them (1, 2, 3) in order of those you see as making it the easiest to seek help for mental health issues.

| Facilitator | Rankings | TOTAL |
| --- | --- | --- |
| **Being aware of your feelings and finding it easy to express them** | 3 3 2 3 1 2 1 1 1 | **19** |
| **Already knowing a health professional quite well (e.g., counsellor, doctor)** | 3 3 2 1 1 1 2 3 1 2 3 2 | **24** |
| Coach has a positive attitude to seeking help | 2 3 2 2 3 3 | 9 |
| Teammates have a positive attitude to seeking help | 3 | 3 |
| Friends have a positive attitude to seeking help | 2 | 4 |
| Family have a positive attitude to seeking help | 3 | 1 |
| **Other people have a positive attitude to seeking help [pooled data: coach, friends, teammates, family]** |  | **17** |
| Other people’s encouragement to seek help | 1 | 3 |
| The staff (e.g., receptionist, doctor, counsellor) are friendly and approachable | 1 1 3 1 | 10 |
| All athletes were required to see a counsellor or psychologist as part of their preparation and training? | 1 | 3 |
| Anonymous access to information and help (e.g., the internet) | 2 | 2 |
| Other (please explain)  *Information* | 2 | 2 |

***Scoring:*** 1=3, 2=2, 3=1, numbers added for total score; Higher scores = more higher ranked votes. Note: Sheets filled out incorrectly, and any ranks over 3, were omitted. On 2 sheets, ranks were included for all facilitators but not in order, e.g., five were given a 1, four were given a 2, and 1 was given a 3.
